# Exploring Risk and Protective Factors for Cyberbullying and Their Interplay: Evidence from a Sample of South Korean College Students

**DOI:** 10.3390/ijerph182413415

**Published:** 2021-12-20

**Authors:** Seong-Sik Lee, Hyojong Song, Jeong Hyun Park

**Affiliations:** 1 Department of Information Sociology, Soongsil University, Seoul 06978, Korea; ss824@ssu.ac.kr; 2 Department of Sociology, Korea University, Seoul 02841, Korea; cusekorea20107@korea.ac.kr

**Keywords:** cyberbullying, morality, self-control, cyberbullying victimization, cyberbullying peers

## Abstract

This study explored risk and protective factors for cyberbullying perpetration and examined whether they independently and interactively predicted cyberbullying perpetration. Based on key propositions of micro-level theories of crime and delinquency, we adopted two risk factors, cyberbullying victimization and association with cyberbullying peers, and two protective factors, morality and self-control. Using a sample of South Korean college students (*N* = 244; 112 women (45.9%), 132 men (54.1%); Mean (age) = 22), we found that the two risk factors were positively associated with cyberbullying perpetration, while only one of the two protective factors, which is morality, had a negative relationship with cyberbullying perpetration. In addition, the two protective factors partially buffered the effects of both risk factors on cyberbullying perpetration. The implications and limitations of these findings were also discussed.

## 1. Introduction

As the recent influence of the online domain on our daily lives has increased dramatically, new types of crimes or deviant behavior based on online or information technology have increasingly appeared. As one form of deviant online behavior, cyberbullying has been increasing along with the growth in the use of mobile devices and online social networking services. According to a recent nationally representative survey on cyberbullying among approximately five thousand U.S. adolescents [1], as of 2019, 36.5% of students reported cyberbullying victimization during their lifetime, and 17.4% reported that they had been cyberbullied within the previous 30 days. Regarding cyberbullying perpetration, 14.8% of students admitted that they had perpetrated cyberbullying during their lifetime, and 6.3% reported having done so in the last 30 days. In South Korea, a nationwide survey with 6279 respondents, including 4779 students and 1500 adults, reported that 33.5% of respondents experienced cyberviolence in 2019, which increased from 32.8% in 2018 and 26% in 2017 [2].

Several studies indicate that, although cyberbullying is primarily in the form of an indirect type of aggression, for example, verbal aggression, its adverse effects on victims, such as delinquency, depression, suicidal ideation, and other behavioral/mental problems, are as significant as traditional bullying, which can harm victims’ physical bodies and inflict physical distress [3,4,5,6,7,8,9]. Moreover, such negative consequences can potentially be more harmful than those of traditional bullying victimization given that cyberbullying via mobile instant messenger services has become more prevalent recently due to the increasing use of smartphone devices. In other words, because perpetrators and victims are connected nearly 24/7 in such a setting, the environment enables perpetrators to cyberbully victims more frequently and more constantly, which, in turn, may lead to more adverse effects on victims [10,11,12].

Given that, in general, bullying tends to occur in a school context, most cyberbullying studies to date have focused on cyberbullying among school-aged teenagers. However, as online communication has begun to increase not only in the school context but also in other settings in general, cyberbullying can be a social problem not only for adolescents in a school context but also individuals in various social and demographic groups as well. In South Korea, for example, adults report even more cyberviolence experiences compared to adolescents; according to the national survey on cyberviolence in 2019, more than a half of adult respondents (54.7%) reported that they experienced either cyberviolence perpetration or victimization, while only 26.9% of student respondents did. More specifically, 32.5% and 48.5% of adults experienced cyberviolence perpetration and victimization respectively, while 18% and 19% of students reported them, respectively [2]. Nevertheless, cyberbullying and online aggression among adults have rarely been examined to date (however, see [13,14,15]). Therefore, more research on cyberbullying in non-school contexts is needed.

In addition, there is a need for research focusing on examining interactive mechanisms of risk and protective factors for cyberbullying based on theories of crime and deviance although many prior studies have explored probable risk and protective factors for cyberbullying and examined their associations with cyberbullying. Some cyberbullying studies have examined the applicability of theoretical explanations for cyberbullying derived from major criminological theories, such as self-control, learning, and strain theories [14,16,17,18]. However, less attention has been paid to how these theoretical variables interact with one another, that is, whether their effects on cyberbullying decrease or increase under certain conditions (however, see [19,20]). Drawing on prior empirical findings as well as the propositions of major criminological theories, we first examined the significance of several probable risk and protective factors for cyberbullying. We also examined how they interact, more specifically, whether protective factors significantly reduce the criminogenic effects of risk factors on cyberbullying. To do so, we used survey data collected from South Korean college students on their smartphone use and cyberbullying experiences.

### 1.1. Risk Factors: Cyberbullying Victimization and Cyberbullying Peers

There are several important risk factors for cyberbullying that can be suggested by major criminological theories. These risk factors may include victimization as a source of an individual’s strain (general strain theory) and differential association with delinquent peers (differential association/social learning theories).

According to general strain theory [21], strain derived from negative life events that individuals experience can be a cause of their criminal and delinquent behavior. When people experience negative life events, they are more likely to experience negative affective states, and crime is one coping strategy that they adopt to release their strain and negative emotions. Empirical findings show that some sources of strain, such as conflict with parents or peers and poor academic performance, are closely associated with negative emotions, including anger, depression, frustration, and fear, and these negative affective states eventually lead to delinquency [22,23,24,25]. The proposed criminogenic mechanism can also be applied to cyberbullying. That is, those exposed to the sources of strain may cyberbully others to cope with their strain and negative emotions. The unique attributes of cyberspace, such as the lack of contact and the anonymity, can be perceived as attractive for coping with one’s stress and negative emotions. For example, Patchin and Hinduja [17] found that both strain and negative emotions, such as anger and frustration, were significantly associated with cyberbullying as well as traditional bullying.

In terms of the effects of strain, Agnew [22,25,26] suggested that some types of strain are more criminogenic than others, particularly when they are seen as unjust, high in magnitude (e.g., severity, frequency, or duration), associated with low social control, and provide some incentive or pressure for criminal coping. Given these attributes of criminogenic strain, criminal or peer victimization was taken as an example of the criminogenic strain, which is assumed to be more likely to be associated with one’s negative emotions and delinquent coping. Thus, if an individual is victimized, they are likely to feel negative emotions, which, in turn, may lead them to perpetrate cyberbullying as a means of retaliating for their victimization and coping with their negative emotions derived from that victimization. Many cyberbullying studies have consistently reported that criminal or peer victimization is significantly associated with cyberbullying perpetration [7,13,15,27,28,29].

In addition to victimization, the association with cyberbullying peers can be another risk factor. According to differential association/social learning theories [30,31], delinquency is learned by others, especially when they have experiences related to crime or rule violations. As an individual frequently socializes with delinquent peers, he or she learns favorable definitions and techniques of crime from those delinquents, accepts pro-criminal attitudes, and can experience reinforcement of conformity to the delinquent peers and peer groups. These social learning processes may eventually lead the person to become involved in crime and delinquency. Cyberbullying perpetration can thus be encouraged by cyberbullying peers, as it is perceived as cool and rewarded among these delinquent peers.

In line with this proposition, prior studies found that association with delinquent peers was an important predictor of online deviant behavior as well [32,33], and recent cyberbullying studies also reported that the effects of delinquent peers on cyberbullying perpetration were significant [16,18]. Furthermore, because cyberbullying involves verbal and indirect aggression, it tends to be perceived less seriously than other deviant behaviors; hence, it is likely that a favorable definition of cyberbullying is more easily accepted through the social learning mechanism. Therefore, differential association with delinquent peers, including cyber-deviant peers, can also be an important risk factor for cyberbullying perpetration.

### 1.2. Protective Factors: Morality and Self-Control

In addition to risk factors for cyberbullying, protective factors for cyberbullying must also be discussed that can prevent someone from engaging in cyberbullying offenses despite the individual being exposed to risk factors. These protective factors may be directly associated with a lower risk of committing cyberbullying. In addition, they may also condition the effects of risk factors for cyberbullying, buffering the criminogenic effects of strain and delinquent peers.

One protective factor of cyberbullying is morality. If one’s morality is high, they are less likely to commit cyberbullying. Morality has to do with whether a person perceives crime as an alternative when they are exposed to criminal opportunities. People with a high level of morality believe that crime is never the right approach and cannot be an alternative regardless of pressure and opportunistic temptation to commit crime; thus, morality prevents them from breaking the rules and laws. However, if an individual has weak moral beliefs, crime can be perceived as an alternative when it is seen as attractive [34,35,36]. Research findings also show that the protective effect of morality on criminal and delinquent behaviors is significant [37,38,39]. Cybercrime is also likely to be perceived as an alternative to weak morality [40]. Similarly, some studies have reported that moral attitudes toward cyberbullying are closely related to cyberbullying perpetration [41,42].

An individual’s self-control can be another important protective factor for cyberbullying. Gottfredson and Hirschi’s general theory of crime [43] focuses on the fact that most crimes are committed impulsively and spontaneously. They pointed out that individuals’ self-control as an ability to control immediate gratification and impulsivity was a cause of crime. That is, people with high self-control are more likely to behave with a long-term perspective for achievement and success rather than pursuing immediate gratification and pleasure, which prevents deviant behaviors. The protective role of self-control can also be applied to deviance in cyberspace, and research findings suggest that self-control is an important protective factor for several types of cybercrime [44,45,46]. Because cyberspace provides a setting in which people behave more impulsively without deliberation due to anonymity and the lack of physical contact, it can be more difficult for people with low self-control to manage their temper and impulsive behavior, which may lead them to commit deviant behavior more easily in cyberspace environments. Furthermore, compared to other types of offense, violence is more closely related to impulsivity rather than being behavior based on a deliberated plan. Self-control is thus an important protective factor for cyberbullying, one form of cyberviolence [14,15,47,48].

### 1.3. Interplay between Risk and Protective Factors for Cyberbullying

Although each of the major theories of crime and delinquency suggests their own risk and protective factors, there is also increasing attention on whether these factors interact. Thus, a factor may have different effects on crime depending on the influence of other factors. Cyberbullying can also be understood by considering the interplay between risk and protective factors, which indicates the conditions that increase the likelihood of cyberbullying perpetration. This approach is based on recent efforts toward theoretical integration focusing on the interplay between risk and protective factors, which has been discussed in general strain theory [22,25] and situational action theory [34,35,36].

According to general strain theory, for instance, strain is suggested to be a cause of crime, but its effect on crime can be moderated by other variables, such as self-control, social bonds, and association with delinquent peers [49,50,51,52]. Situational action theory also suggests that several types of interaction effects can be examined to understand the mechanism of crime, considering that both person and environment interact with one another. For example, associating with delinquent peers indicates an environment in which weak moral rules are justified and even rewarded, but its criminogenic effects may not be identical between those with weak morality and those with strong morality. In other words, an individual’s moral disengagement may enhance the effect of delinquent peers, while it can be buffered by one’s high morality. In addition, it has been reported that the negative effect of criminogenic environments and self-control interact [53,54,55], which is the case for cyber-deviance as well [32]. Therefore, the likelihood of cyberbullying perpetration increases when an individual has delinquent peers and low self-control, while it decreases when their self-control is high.

### 1.4. Current Focus

The current study thus aims to examine the interactions between risk and protective factors for cyberbullying as well as their independent associations with cyberbullying. Risk factors refer to a necessary condition that directly triggers cyberbullying perpetration, including strain and association with delinquent peers at the initial stage, while the protective factors are an individual’s propensity to reduce the effects of these motivators. Therefore, we can expect that individuals with risk factors for cyberbullying are more likely to cyberbully others, and among the risk group, those with protective factors are less likely to commit cyberbullying than those with no protective factors, as the effects of risk factors are attenuated by the protective factors.

As previously discussed, both cyberbullying victimization and association with cyberbullying peers can be considered risk factors for cyberbullying perpetration, while both morality and self-control can be considered protective factors. Thus, our three research hypotheses are as follows:

**Hypothesis** **1.** *The two risk factors for cyberbullying, cyberbullying victimization and association with cyberbullying peers, predict an increased likelihood of cyberbullying perpetration.*

**Hypothesis** **2.** *The two protective factors for cyberbullying, morality and self-control, predict a decreased likelihood of cyberbullying perpetration.*

**Hypothesis** **3.** *The two protective factors for cyberbullying buffer the effects of the risk factors for cyberbullying.*

## 2. Participants and Methods

### 2.1. Particiapants

One of the primary foci in the current study was to examine whether the proposed mechanism, which is the interplay between risk and protective factors for cyberbullying, is applicable to young adults. Therefore, in 2017, we conducted a survey of 266 South Korean college students from seven randomly selected colleges in Seoul, South Korea. Drawing on the quota-sampling method, we recruited participants based on the following criteria: (1) assign 30 to 40 participants to each university and (2) balance the ratios of gender (males to females) and major (the humanities/social sciences to engineering/natural sciences). Out of 266 students, 115 and 143 were women and men, respectively, while eight declined to report their gender. Regarding participants’ majors, 135 selected their majors in the humanities or social sciences, 96 in engineering or natural sciences majors, and 22 in arts or sports. Thirteen participants declined to report their majors. The average age of original participants was approximately 22 years. Through the data-cleaning process, 22 incomplete responses, such as those with missing information regarding gender, age, or cyberbullying perpetration, were excluded; thus, a total of 244 responses were finally used for data analysis. The final sample consists of 132 men (54.1%) and 112 women (45.9%). Their ages ranged from 18 to 27 years, and the average age was 22. For their family socioeconomic status, 125 participants reported that their family held the “middle” socioeconomic status, which is more than the half (51.2%), followed by “high” (30.3%), “low” (11.5%), “extremely high” (4.5%), and “extremely low” (see Table 1).

### 2.2. Measures

*Cyberbullying perpetration*. Cyberbullying perpetration is the dependent variable of this study. Six items were created and asked based on Willard’s study [56] and the cyber-violence survey conducted by the Korea Internet and Security Agency. Respondents were asked the following: “For the last year, through your smartphone, have you (1) committed verbal abuse against and humiliated someone else, (2) spread rumors about someone else, (3) spread private information about someone else, (4) stalked someone else by continually sending them emails or texts and posting comments on their blogs or Facebook pages, (5) sexually harassed someone else by sending obscene photos and video clips, or (6) ostracized someone else in a group chat room while using mobile instant messengers?” Participants responded to the items with five response options, “not at all”, “once”, “two to three times”, “four to nine times”, and “ten or more times”. Each response was scored from 0 to 4, and the sum of the scores was used (alpha = 0.890).

*Cyberbullying victimization.* As one of the risk factors, cyberbullying victimization was measured with the same six items as those for cyberbullying perpetration mentioned above, with the items modified to cyberbullying victimization. The same five response options were applied, and the sum of the scores was also used (alpha = 0.811).

*Association with cyberbullying peers.* The other risk factor, association with cyberbullying peers, was measured with the same six items as those used for cyberbullying perpetration as mentioned above, but the items were modified, asking respondents if they had any cyberbullying peers who were engaged in those offenses and, if so, how many. Five response options were applied: “none”, “one”, “two to three peers”, “four to nine peers”, and “ten or more peers”. Scores from 0 to 4 were assigned to five responses (alpha = 0.867).

*Morality*. As one of the protective factors, an individual’s morality regarding cyberbullying perpetration was measured with six items related to whether respondents would think that the six forms of cyberbullying perpetration mentioned above are morally wrong. A five-point Likert scale was used, with the responses “strongly disagree” (=1), “disagree” (=2), “neutral” (=3), “agree” (=4), and “strongly agree” (=5), and the sum of the scores for all six items was used (alpha = 0.957).

*Self-control*. The other protective factor, self-control, was measured using a modified and shortened version of the Grasmick and colleagues’ scale [57]; as applied in a previous study [58], a single indicator was used to measure each of the six aspects of low self-control traits, such as impulsivity, risk-seeking, simple task, physical activity, self-centeredness, and volatile temper; thus, a total of six items were applied, and those six items were measured through a five-point Likert scale, ranging from “strongly disagree” (=1) to “strongly agree” (=5). The scores for the six items were reversed and added (alpha = 0.632) so that the higher the scores, the higher the self-control.

*Control variables*. Finally, *gender*, *age*, and *perceived family socioeconomic status* were controlled for in all analytic models. For gender, men and women were coded as 0 and 1, respectively. Respondents were asked for the year of birth, which was then recoded as their age. Regarding perceived family socioeconomic status, respondents were also asked to assess their family income and to select a response from among “extremely low” (=1), “low” (=2), “middle” (=3), “high” (=4), and “extremely high” (=5). Table 1 displays the descriptive statistics of the variables in this study.

### 2.3. Plan of Analysis

To examine the three research hypotheses, we applied three ordinary least square regression models. First, the two risk factors, cyberbullying victimization and cyberbullying peers, were included in Model 1 along with the control variables, such as gender, age, and perceived family socioeconomic status, to examine whether both risk factors independently and significantly increase the likelihood of perpetrating cyberbullying. In Model 2, we then added the two protective factors, morality and self-control, to Model 1 to examine whether both protective factors independently and significantly reduce the likelihood of perpetrating cyberbullying. In Model 3, we created and added four interaction terms using the two risk and two protective factors, (1) cyberbullying victimization * morality, (2) cyberbullying victimization * self-control, (3) association with cyberbullying peers * morality, and (4) association with cyberbullying peers * self-control, to examine the proposed buffering role of protective factors in the associations between risk factors and cyberbullying perpetration. For interaction terms, each risk and protective factor was mean centered to reduce multicollinearity. All statistical analyses were executed using Stata 15.

## 3. Results

Table 2 shows the three different models for cyberbullying perpetration. In Model 1, the independent associations of the two risk factors with cyberbullying perpetration controlling for sociodemographic variables, including gender, age, and perceived family SES, are reported. As shown in Model 1, both cyberbullying victimization and association with cyberbullying peers significantly increased the likelihood of cyberbullying (*p* < 0.01). The standardized coefficient of the variable of cyberbullying victimization (B = 0.493) was larger than that of association with cyberbullying peers (B = 0.319), and approximately 59.9% of the variance was explained by the two risk factors and the three control variables (R^2^ = 0.599).

Model 2 includes the two protective factors, morality and self-control, in addition to the variables in Model 1. In Model 2, both risk factors remained significant despite the addition of the two protective factors (*p* < 0.01), and the standardized coefficient for the variable of cyberbullying victimization (B = 0.482) was also larger than that of association with cyberbullying peers (B = 0.315). Regarding the independent associations of protective factors with cyberbullying perpetration, higher morality was significantly associated with a lower chance of cyberbullying (B = −0.118, *p* < 0.01), while self-control was not significantly associated with cyberbullying (B = 0.055, ns). The explained variance of the model increased slightly to 61.4% (R^2^ = 0.614) compared to Model 1.

**Figure 1:**
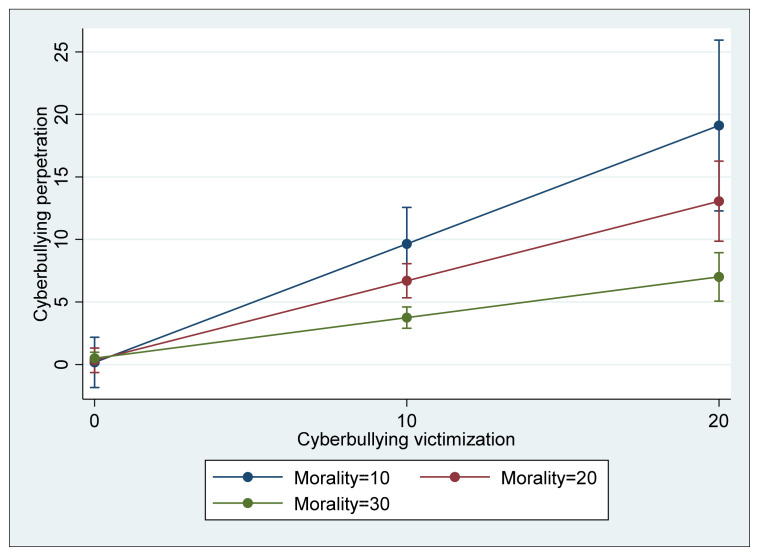
Interaction between cyberbullying victimization and morality predicting cyberbullying perpetration (with 95% confidence intervals).

**Figure 2:**
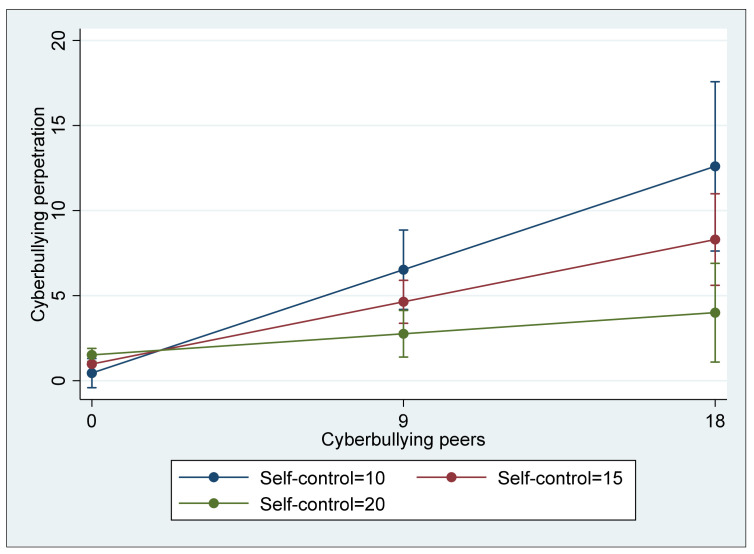
Interaction between association with cyberbullying peers and self-control predicting cyberbullying perpetration (with 95% confidence intervals).

In Model 3, four different interaction terms between the risk and protective factors were included to examine whether the protective factors significantly reduce the effect of risk factors on cyberbullying perpetration. As in the two previous models, cyberbullying perpetration was significantly and positively associated with the two risk factors, cyberbullying victimization (B = 0.427, *p* < 0.01) and association with cyberbullying peers (B = 0.189, *p* < 0.01). Similar to Model 2, morality was significantly and negatively associated with cyberbullying (B = −F0.097, *p* < 0.05); however, self-control was insignificant (B = 0.018, ns). Regarding the four interaction terms, two terms, cyberbullying victimization * morality and association with cyberbullying peers * self-control, showed a negative direction, and they were statistically significant at *p* < 0.01. That is, morality in addition to its direct association with cyberbullying perpetration significantly buffered the direct effect of cyberbullying victimization on cyberbullying (see Figure 1), and self-control was also a significant moderator that buffered the direct effect of association with cyberbullying peers (see Figure 2) although its direct association with cyberbullying perpetration was not significant. In terms of the explained variance of Model 3, the model accounted for 65.6% of the total variance (R^2^ = 0.656), a 4.2 percent increase from the explained variance of Model 2, which is a significant increase (ΔR^2^ = 0.042, F(4,232) = 7.03, *p* < 0.05).

## 4. Discussion

The current study focused on examining whether risk and protective factors suggested by some criminological theories account for cyberbullying perpetration among South Korean young adults. Furthermore, this study also examined whether the criminogenic effects of risk factors, cyberbullying victimization, and association with cyberbullying peers were significantly buffered by protective factors, specifically morality and self-control.

Consistent with Hypothesis 1, the two risk factors, cyberbullying victimization and association with cyberbullying peers, were positively correlated with cyberbullying even when controlling for the protective factors as well as other control variables. That is, college students who were more cyberbullied and had more cyberbullying peers were more likely to perpetrate cyberbullying. The findings imply that the suggested mechanisms of deviant behavior derived from general strain theory and differential association/social learning theories can be applied to cyberbullying as well. As suggested by general strain theory, cyberbullying victimization can be perceived as a more direct, unfair, and serious negative life experience for cyberbullying victims compared to other types of negative life events that heighten one’s strain. This, in turn, may lead them to negative affective states such as anger and frustration and eventually result in cyberbullying perpetration. Association with cyberbullying peers can also lead to cyberbullying, as suggested in differential association/social learning theories, as individuals learn a favorable definition and techniques of cyberbullying from their peers, and they want to be recognized and accepted by their cyberbullying peers for their cyberbullying perpetration. This suggests that the risk factors in this study play an important role as a necessary condition for cyberbullying and that we should consider how to decrease the negative influence of cyberbullying victimization and cyberbullying peers, particularly in regard to developing effective policies to reduce cyberbullying perpetration. As general strain theory suggests, for instance, cyberbullying victimization may lead to high levels of strain and negative feelings closely related to deviant behaviors. Thus, implementing counseling programs that can help cyberbullying victims cope with their strain derived from their victimization in legitimate and healthy ways and developing educational programs on effective techniques or tips for cyberbullying prevention are recommended. In addition, we should consider adopting intervention programs designed to block the differential association and social learning processes for those who reported associating with cyberbullying peers as well as for their cyberbullying peers.

In terms of Hypothesis 2, our findings indicate that only one of the protective factors, morality, had a significantly negative relationship with cyberbullying perpetration, while the other one, self-control, did not have a significant association with the dependent variable. In other words, college students with higher morality on cyberbullying were less likely to perpetrate cyberbullying, but their levels of self-control seemed to be irrelevant to preventing their cyberbullying behavior or, at least, its independent association. Regarding morality, young adults who believed that cyberbullying was wrong were less likely to perpetrate cyberbullying regardless of variations in other risk factors, such as cyberbullying victimization and association with cyberbullying peers. Thus, we can see the possibility that the protective mechanism of moral belief may be applicable to cyberbullying as well as being an important protective factor for other forms of delinquency. In terms of the insignificancy of self-control, it may imply that the expected protective mechanism of self-control is not applicable to cyberbullying perpetration. That is, self-control is expected to prevent many forms of delinquency as it allows individuals to deliberate and pursue long-term consequences and goals rather than seeking after short-term gratification or immediate pleasure that can be fulfilled by delinquency. Therefore, the insignificant association between self-control and cyberbullying may be partially derived from how serious people would perceive cyberbullying in that cyberbullying may be taken less seriously compared to other traditional forms of delinquency. In other words, since cyberbullying is often not a form of physical attack or fight, young adults, even those with higher self-control, may perceive it as a minor or trivial deviance that rarely ensues any adverse consequences, such as official punishment and informal social disadvantages. Thus, some educational programs targeting people’s perception of cyberbullying may be recommended, for example, for those aiming to change their perception that cyberbullying is not trivial and can be more harmful than traditional bullying, and thus, more severe responses should follow.

As for Hypothesis 3, two protective factors, higher morality and higher self-control, were shown to play a moderating role, as expected, partially buffering the criminogenic effect of cyberbullying risk factors. Specifically, it was found that morality buffered the effect of cyberbullying victimization on cyberbullying perpetration, while self-control played a role in attenuating the effect of association with cyberbullying peers. From the perspective of criminological theories, this is consistent with the aspect that is emphasized in the integrated theories of crime, such as general strain theory and situational action theory: an individual’s criminal behavior can be better explained through the interactions between risk factors derived from environmental and contextual settings and protective factors based on the individual’s traits. That is, the mechanisms of an individual’s criminal behavior can be specified as depending on the circumstances/context and individual traits that cause an increase or decrease in the likelihood of one’s involvement in crime. Although cyberbullying is often committed by those who were cyberbullied themselves because their victimization can be a source of criminogenic strain, the possibility of being involved in cyberbullying can be minimized when a cyberbullying victim has a strong moral belief that cyberbullying others is wrong, as it buffers the criminogenic effect of strain derived from his or her cyberbullying victimization. In addition, although association with cyberbullying peers increases the likelihood of cyberbullying perpetration, it can also be decreased when an individual with cyberbullying peers has a high level of self-control, which is an individual’s ability to restrain their impulsive behavior in regard to immediate or short-term gratification, such as cyberbullying, as it can be a means to immediately reduce their stress given that self-control buffers the criminogenic effect of association with cyberbullying peers. These findings have both practical and theoretical implications. Regarding an effective future response to cyberbullying perpetration, although some policies that help potential perpetrators not to be exposed to the risk factors should be prioritized, it is also important to consider policies that strengthen one’s traits related to morality, conscience, deliberation, and self-constraint. For instance, we should consider providing more resources for educational programs on cyber ethics, which is expected to enhance one’s morality in cyberspace. In addition, we should also consider expanding programs or interventions that help parents provide their children with effective parenting with affection, consistency, and discipline, which is eventually associated with children’s development of self-control.

## 5. Conclusions

The current study found that the two proposed risk factors, cyberbullying victimization and association with cyberbullying peers, were significantly and positively related to cyberbullying perpetration, while only one of the two proposed protective factors, morality, was significantly and negatively associated with cyberbullying perpetration as expected. Self-control was not a significant protective factor. As for the proposed buffering role of protective factors, our findings partially supported it. Morality successfully attenuated the criminogenic effect of cyberbullying victimization but not for that of association with cyberbullying peers. On the other hand, self-control significantly buffered the effect of cyberbullying peers, while it did not play a buffering role for the relationship between cyberbullying victimization and cyberbullying perpetration. Our findings show that not only those risk and protective factors but their interactions as well play an important role in predicting cyberbullying perpetration even in the South Korean context and among young adults. Given that cyberviolence perpetrated by adults is even more prevalent than that by adolescents in South Korea [2], the findings of the present study may be considered when developing more effective strategies for cyberbullying prevention.

The limitations of the current study should also be discussed. First, because the data used in this study are cross-sectional, it is difficult to examine the causality of the cyberbullying perpetration mechanism suggested in this study. Although it was found that some risk and protective factors were influential and some of the interactions between both risk and protective factors were significant, as suggested by theories of crime and delinquency, it is also possible that involvement in cyberbullying affects cyberbullying victimization and association with cyberbullying peers rather than the opposite direction hypothesized in this study. Thus, future studies applying longitudinal data are needed to examine the causal mechanism by which these risk and protective factors and their interactions precede cyberbullying perpetration.

Second, although the present study shows the results of non-Western young adults, who have been relatively less studied to date, which is the strength of the current study, the features of the participants in this study may have some limitations related to external validity. That is, because the participants only included college students who were actively enrolled at seven colleges in Seoul, South Korea, it is not representative of South Korea’s entire population of young adults. In other words, the sample did not include groups such as those who did not live in Seoul and those who did not go to college. Thus, the theoretical implications of the current study may be limited, and the policy implications may not be applicable to these groups who were excluded from the sample. Therefore, we anticipate future studies with more representative samples, particularly for non-Western young adults.

## Figures and Tables

**Table 1:** Descriptive Statistics.

Variable	Mean	S.D.	Min	Max
*Dependent Variable*				
Cyberbullying perpetration	1.82	3.66	0	21
*Risk Factors*				
Cyberbullying victimization	3.32	4.03	0	20
Cyberbullying peers	1.67	3.06	0	18
*Protective Factors*				
Morality	28.00	4.07	6	30
Self-control	18.38	3.79	9	30
*Control Variables*				
Age	21.98	2.08	18	27
Gender	*N*	*%*		
Female	112	45.9		
Male	132	54.1		
Perceived Family SES	*N*	*%*		
Extremely low	6	2.5		
Low	28	11.5		
Middle	125	51.2		
High	74	30.3		
Extermely high	11	4.5		

*N* = 244.

**Table 2:** Ordinary Least Square Regression Models Predicting Cyberbullying Perpetration.

Variables	Model 1			Model 2			Model 3		
	b	S.E.	B	b	S.E.	B	b	S.E.	B
Cyberbullying victimization (CV)	0.448 **	0.050	0.493	0.437 **	0.050	0.482	0.388 **	0.049	0.427
Cyberbullying peers (CBP)	0.380 **	0.066	0.319	0.375 **	0.066	0.315	0.225 **	0.074	0.189
Morality (MO)	-	-	-	−0.106 **	0.038	−0.118	−0.087 *	0.037	−0.097
Self-control (SC)	-	-	-	0.053	0.041	0.055	0.017	0.039	0.018
CV*MO	-	-	-	-	-	-	−0.031 **	0.012	−0.173
CV*SC	-	-	-	-	-	-	0.021	0.012	0.117
CBP*MO	-	-	-	-	-	-	−0.005	0.016	−0.022
CBP*SC	-	-	-	-	-	-	−0.054 **	0.019	−0.220
Female	−0.752 *	0.315	−0.103	−0.726 *	0.311	−0.099	−0.652 *	0.302	−0.089
Age	−0.139	0.075	−0.079	−0.121	0.074	−0.069	−0.103	0.071	−0.058
Perceived Family SES	0.159	0.188	0.035	0.208	0.187	0.046	0.182	0.180	0.040
Constant	2.57	1.81	-	4.06	2.11	-	4.02	2.04	-
R^2^	0.599			0.614			0.656		

* indicates *p* < 0.05 level. (two-tailed test); ** indicates *p* < 0.01 level. (two-tailed test).

## Data Availability

Not applicable.
